# Influence of Sm^3+^ Ions on the Structural, Optical and Luminescent Properties of Zinc–Antimony–Boro–Germanate Glasses

**DOI:** 10.3390/ma19091885

**Published:** 2026-05-03

**Authors:** Razvan Stefan, Bogdan Golgotiu, Maria Bosca, Raluca Lucacel-Ciceo, Liviu Bolundut, Petru Pascuta

**Affiliations:** 1 Department of Preclinical Sciences, University of Agricultural Science and Veterinary Medicine, 3-5 Calea Manastur, 400372 Cluj-Napoca, Romania; rstefan@usamvcluj.ro; 2 Life Sciences Institute, 3-5 Calea Manastur, 400372 Cluj-Napoca, Romania; 3 Department of Physics and Chemistry, Technical University of Cluj-Napoca, 103-105 Muncii Avenue, 400641 Cluj-Napoca, Romania; bogdan.golgotiu@phys.utcluj.ro (B.G.); maria.bosca@phys.utcluj.ro (M.B.); 4 European University of Technology, European Union; 5 Faculty of Physics, Babes-Bolyai University, 400084 Cluj-Napoca, Romania; raluca.lucacel@ubbcluj.ro; 6 Institute for Interdisciplinary Research in Bio-Nano-Sciences, Babes-Bolyai University, 400271 Cluj-Napoca, Romania

**Keywords:** samarium-doped glass, luminescence data, XRD, XPS, DR-UV-Vis

## Abstract

Zinc–antimony–boro–germanate glasses highly doped with Sm_2_O_3_ were synthesized by the conventional melt-quenching method. Their structural, optical, and luminescent properties were systematically investigated by X-ray diffraction (XRD), X-ray photoelectron spectroscopy (XPS), diffuse reflectance UV–Vis (DR-UV–Vis), and photoluminescence (PL) spectroscopy. XRD analysis confirmed the amorphous nature of all prepared samples. XPS measurements were used to examine the surface chemical composition of the Sm_2_O_3_-doped glasses, with particular focus on verifying samarium incorporation and identifying its oxidation state after synthesis, since Sm ions act as the luminescent centers in these materials. For the sample containing the highest Sm_2_O_3_ concentration, the DR-UV–Vis spectrum exhibited ten absorption bands assigned to intra 4f electronic transitions. Based on these data, the nephelauxetic and bonding parameters were determined, indicating that increasing Sm_2_O_3_ content enhances the ionic character of the bonds within the glass network. PL spectra revealed three characteristic emission bands associated with Sm^3+^ luminescent centers. The emission intensity reached a maximum at 3 mol% Sm_2_O_3_, while further increases in samarium content led to luminescence quenching. The most intense emission band was in the yellow–orange region of the visible spectrum, highlighting the potential of these materials for yellow–orange-emitting solid-state laser applications. The excitation spectra show that the optical response is strongly dependent on concentration, with a sample doped with 3 mol% Sm_2_O_3_ exhibiting the highest excitation efficiency. The dominant excitation band centered near 402 nm, together with weaker bands in the blue region, indicating that these glasses are promising candidates for near-UV-pumped orange-emitting photonic devices.

## 1. Introduction

Glasses and glass ceramics containing rare earth (RE) ions, such as Sm^3+^, have been extensively investigated because of their importance as functional materials for a wide range of applications, including optical amplifiers, solid-state lasers, display technologies and other photonic devices [1,2,3,4,5,6,7,8,9,10].

Zinc presence enhances the magnetic, electrical, mechanical resistance and optical properties of both glasses and glass ceramics [11]. Moreover, zinc is environmentally benign and exhibits low toxicity, which supports its use in bioactive glasses, particularly because of its antibacterial effect [12]. ZnO improves the chemical durability of the glass samples due to its non-hygroscopic nature [13]. Since Zn^2+^ ions possess a filled d-shell, they do not show d–d transitions; nevertheless, oxygen-to-zinc charge-transfer bands occur in the ultraviolet spectral range, suggesting that glasses that contain zinc are promising materials for UV-emitting laser applications [14].

Antimony is a multivalent element and exists in glass systems predominantly in the +3 and +5 oxidation states because of its five valence electrons. In glass matrices, Sb^3+^ ions may act as luminescent centers, and usually give rise to four emission bands, and can also act as sensitizers for lanthanide emission, inducing up-conversion of RE ions. In these cases their characteristics are strongly influenced by the local structure of the glass network [15,16].

Only limited information is available in the literature regarding antimony-related luminescence. Usually, antimony is introduced in the glass as an additive in order to improve host properties for applications in optical devices, power limiters, and optical amplifiers [17,18].

Boron (III) oxide was selected as one of the glass formers because it is a classical network former that allows the preparation of transparent glasses at relatively low melting temperatures, while offering good thermal stability and a high ability to accommodate RE ions [19].

The suitability of glasses containing B_2_O_3_ for optical applications has also been demonstrated in related borate and boro–zinc tellurite systems, where optical, physical, and structural improvements were reported [20,21]. However, pure borate glasses generally exhibit relatively high phonon energy, which may enhance multiphonon relaxation and thereby reduce the luminescence efficiency of RE activators [22]. To limit this drawback, GeO_2_ was added to the glass network, since germanate-based glasses are well known for their lower phonon energy, good thermal and chemical durability, high refractive index, and excellent spectroscopic behavior, desirable for photonic applications [22,23,24,25].

Recent studies on bismuth–germanate–germanium glasses have highlighted their potential for optical signal amplification, broadband luminescence, and thermally stable infrared-emitting materials [23,24,25].

The advantages of the B_2_O_3_ and GeO_2_ glass former combination, in boro–germanate glasses, provides a suitable compromise between good glass-forming ability, broad optical transparency, high RE solubility, and a low level of non-radiative losses [22,26,27,28]. This choice is further supported by previous investigations on RE-doped boro–germanate glasses, where promising structural, thermal, and luminescence properties have been found for Tb^3+^-, Dy^3+^-, and Eu^3+^-activated glasses [26,27,28].

These make boro–germanate glass an appropriate host for Sm^3+^ ions, because the emission efficiency of these ions strongly depends on the local environment and on the suppression of non-radiative decay.

Indeed, lanthanum boro–germanate glass doped with Sm^3+^ ions exhibits intense reddish-orange emission and relatively large, stimulated emission cross-sections, confirming the suitability of this samarium-doped glass family as photonic materials in the visible range [29].

Among the RE ions, samarium is of particular interest because it exhibits intense luminescence, especially in the orange–red domain of the visible spectrum. These optical properties make samarium-doped glasses functional for laser applications, optical communication systems, high-density data storage, and display devices [30].

In addition, the luminescence of Sm^3+^ ions can be further enhanced by co-doping the glass matrix with silver or copper nanoparticles [31,32,33].

Motivated by the complementary advantages of borate, germanate, antimonate, and zinc-containing glass networks, together with the remarkable orange–red luminescence of Sm^3+^ ions, this study was undertaken to develop new photonic materials with potential for visible-emitting optical applications. The selected multicomponent glass system is expected to combine high thermal and chemical stability, favorable optical characteristics, and an appropriate local environment for efficient RE incorporation and emission. Therefore, the present work focuses on the preparation of novel glass samples with composition xSm_2_O_3_·(100 − x)[2GeO_2_·2B_2_O_3_·Sb_2_O_3_·ZnO] and on the systematic investigation of their structural and optical properties by X-ray diffraction (XRD), X-ray photoelectron spectroscopy (XPS), diffuse reflectance UV–Vis (DR-UV–Vis), and photoluminescence.

## 2. Materials and Methods

Samples belonging to the novel xSm_2_O_3_·(100 − x)[2GeO_2_·2B_2_O_3_·Sb_2_O_3_·ZnO] system (0 ≤ x ≤ 15 mol%) were synthesized from high-purity Sm_2_O_3_, GeO_2_, B_2_O_3_, Sb_2_O_3_, and ZnO (Alfa Aesar (Ward Hill, MA, USA) and Merck (Darmstadt, Germany)) weighed in stoichiometric proportions. The precursor oxides were thoroughly mixed and ground in an agate mortar until homogeneous batches were obtained. The resulting powders were then transferred to sintered corundum crucibles and melted in an electric furnace at 1200 °C for 5 min. Subsequently, the melts were rapidly quenched to room temperature by pouring them onto stainless-steel plates.

XRD measurements were carried out at room temperature using a Shimadzu XRD-6000 diffractometer (Shimadzu Corporation, Kyoto, Japan) with Cu Kα radiation (λ = 1.54 Å), operated at 40 kV and 30 mA.

XPS measurements were performed on finely powdered samples using a SPECS PHOIBOS 150 MCD system (SPECS Surface Nano Analysis GmbH, Berlin, Germany) equipped with a monochromatic Al Kα X-ray source (250 W, hν = 1486.6 eV), a hemispherical analyzer, and a multichannel detector. The analyses were carried out under ultra-high-vacuum conditions, with the pressure in the analysis chamber maintained in the 10^−8^–10^−9^ mbar range. Surface charging effects during XPS analyses were compensated using an electron gun. All spectra were calibrated against the C 1s photoelectron peak at 284.8 eV. Survey spectra were acquired over the 0–1200 eV binding energy range using a pass energy of 60 eV to identify the surface chemical composition. Due to the significant overlap between the O1s and Sb3d signals, as well as between B1s and Ge3s, reliable quantification of surface atomic concentrations could not be achieved. High-resolution spectra were recorded with an analyzer pass energy of 20 eV and analyzed after subtraction of a Shirley background. Peak deconvolution was carried out using CasaXPS software (Casa Software Ltd., Devon, UK, Version 2.3.26PR1.0), employing GL(30–40) line shapes.

DR-UV-Vis spectra were recorded from MgO pellets on a PerkinElmer Lambda 45 spectrometer (PerkinElmer, Inc., Waltham, MA, USA) equipped with an integrating sphere.

Room-temperature photoluminescence spectra were acquired using an Able Jasco FP-6500 spectrofluorometer with a Xe lamp (JASCO Corporation, Hachioji, Tokyo, Japan) as the excitation source and an excitation wavelength of 402 nm. In addition, excitation spectra were also collected at room temperature.

## 3. Results and Discussion

### 3.1. XRD Data

**Figure 1:**
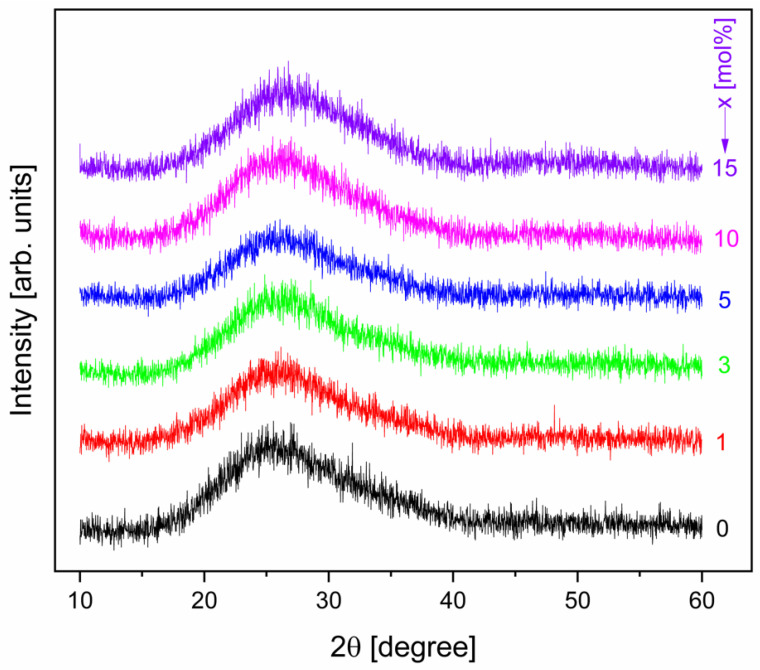
XRD diffractograms of the xSm_2_O_3_·(100 − x)[2GeO_2_·2B_2_O_3_·Sb_2_O_3_·ZnO] system samples.

Figure 1 shows the XRD patterns of the studied glasses recorded in the range of 10° ≤ 2θ ≤ 60°. XRD was used to evaluate the structural order of the samples. All diffraction patterns exhibit a broad diffuse halo centered at approximately 25–30°, with no sharp Bragg reflections. This feature indicates that the samples are predominantly amorphous, with no detectable long-range crystallinity within the instrumental detection limit.

With increasing Sm_2_O_3_ content, only slight changes in the intensity of the broad halo are visible, indicating modifications in the short- to medium-range structural arrangement rather than the formation of crystalline phases. Increasing the Sm_2_O_3_ concentration up to 15 mol% does not induce crystallization or phase separation, which shows a good solubility of Sm^3+^ ions in the zinc–antimony–boro–germanate glass matrix.

### 3.2. XPS Data

X-ray photoelectron spectroscopy (XPS) is one of the most widely used surface analysis techniques for investigating the elemental composition and chemical states of materials through the binding energies (BE) of emitted electrons, with a probing depth of up to approximately 2.5 nm. In the case of luminescent glasses, XPS is particularly useful for assessing the surface chemical environment, since the surface composition and oxidation states may influence defect-related processes and, consequently, the optical and luminescent properties of the material.

**Figure 2:**
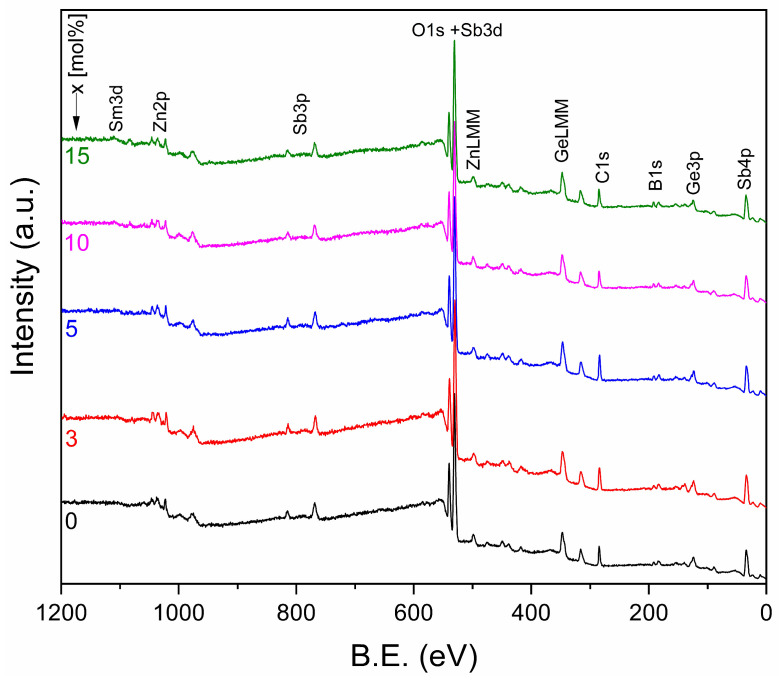
XPS survey spectra of as-prepared samples.

The XPS survey spectra of the synthesized samples, presented in Figure 2, were used to examine their surface composition. Based on the positions and relative intensities of the photoelectron peaks, the presence of O, Ge, B, Sb, Zn, and Sm was identified confirming the successful incorporation of all intended components into the glass composition. Owing to the significant overlap of photoelectron peaks from different elements in the same binding energy region, quantitative determination of atomic concentrations was not carried out.

**Figure 3:**
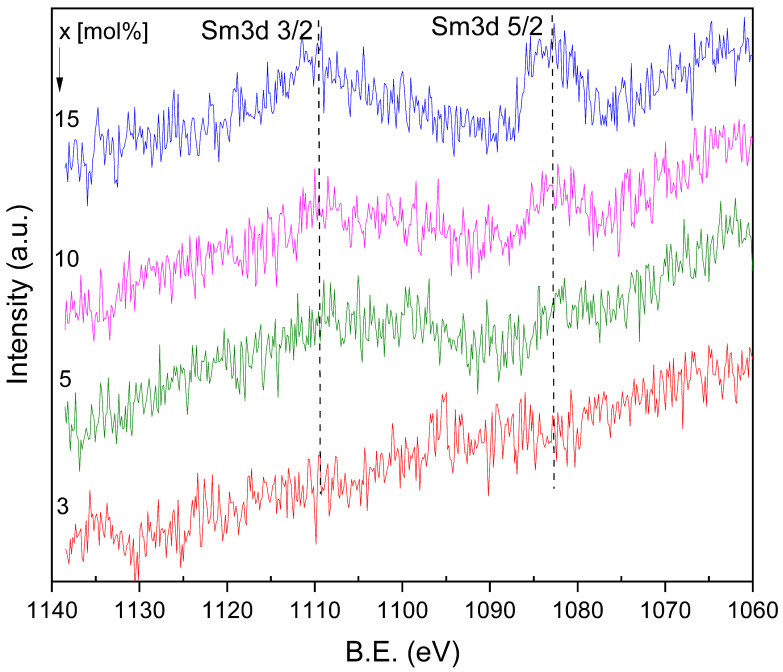
High-resolution XPS spectra of Sm3d.

The high-resolution XPS spectra recorded in the Sm 3d region (Figure 3) exhibit two components centered at about 1083 and 1110 eV, assigned to the Sm 3d_5/2_ and Sm 3d_3/2_ levels, respectively. The observed binding energies are consistent with samarium in the Sm^3+^ oxidation state [34]. These peaks are clearly resolved only for the sample with the highest Sm_2_O_3_ doping level (10 and 15 mol%), while for lower dopant concentrations the weak signal intensity prevents reliable detection. This behavior confirms both the successful incorporation of samarium into the glass composition and its predominantly trivalent state presence.

### 3.3. UV-Vis Data

**Figure 4:**
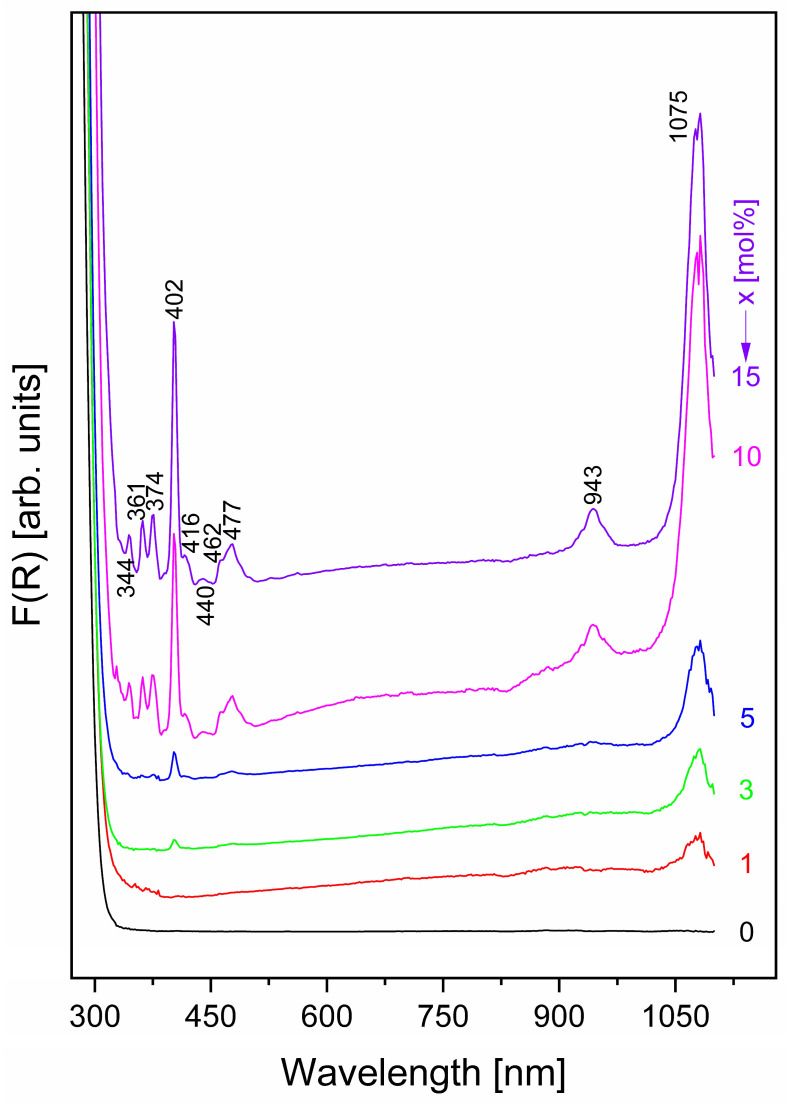
UV-VIS spectra of the xSm_2_O_3_·(100 − x)[2GeO_2_·2B_2_O_3_·Sb_2_O_3_·ZnO] glasses.

Figure 4 presents the UV-vis spectra of the synthesized glass samples, providing insights into their surface composition. All spectra were recorded at room temperature in reflectance using an integrating sphere.

The matrix presents no absorption peaks (Figure 4). The intensity of the peaks increases with the increased concentration of samarium (III) ions in the obtained glass. The sample with the highest concentration of samarium (15 mol% Sm_2_O_3_) presents ten absorption peaks, 344 nm, 361 nm, 374 nm, 402 nm, 416 nm, 440 nm, 462 nm, 477 nm, 943 nm, and 1075 nm, that correspond to different f-f electronic transitions from the ^6^H_5/2_ ground state to different excited states (^4^D_7/2_, ^4^D_3/2_, ^4^L_17/2_, ^6^P_3/2_, ^6^P_5/2_, ^4^G_9/2_, ^4^I_13/2_, ^4^I_11/2_, ^6^F_11/2_, ^6^F_9/2_) in agreement with the literature data [35,36].

**Figure 5:**
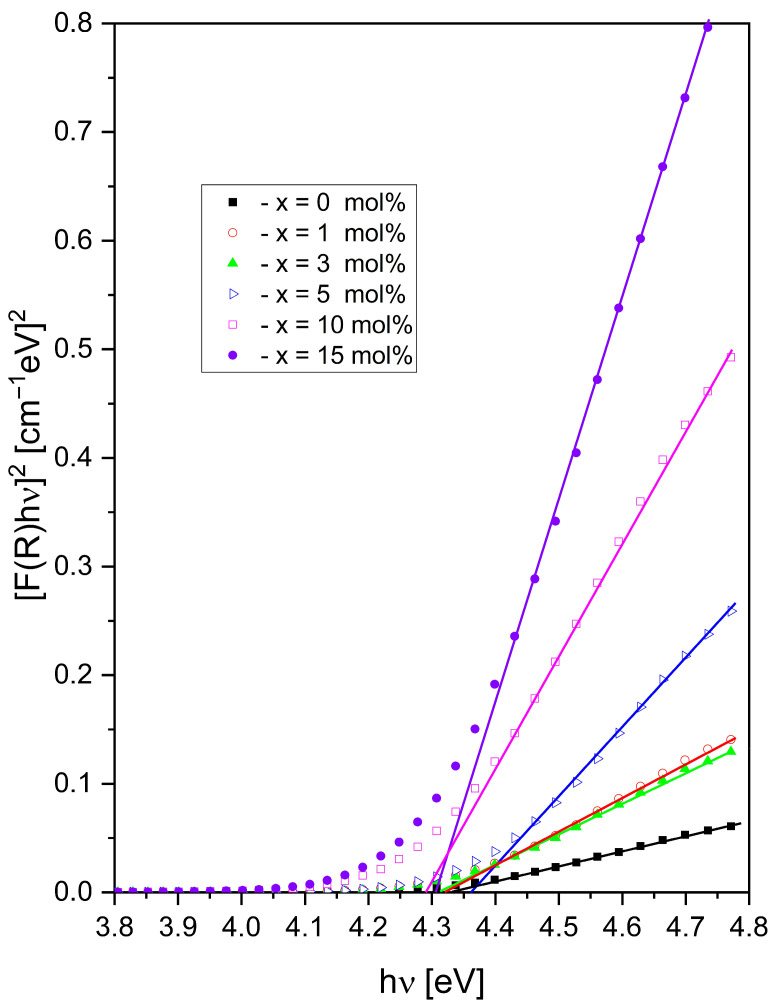
Variation of (F(R)∙hυ)^2^ versus hυ for the studied glasses.

For clarity, Figure 5 presents all absorption peaks observed for the sample containing 15 mol% Sm_2_O_3_ in the batch composition.

Based on these results, the nephelauxetic ratio (β) and bonding parameter (δ) were calculated in order to estimate the ionic or/and covalent character of the bonds in the vitreous network. The method described in the literature [37,38] has been used to calculate β and δ values. Positive values of the bonding parameter δ indicate a predominance of covalent character, whereas negative values suggest a more ionic nature of the bonds in the investigated glass samples. In our case, the δ parameter varies with Sm^3+^ concentration. For the sample containing 1 mol% Sm_2_O_3_, the δ value is 0.401 (positive), indicating a mainly covalent nature of the bond. With increasing Sm^3+^ concentration, δ decreases progressively, reaching −0.047 (negative) for the sample with 15 mol% Sm_2_O_3_, which indicates a shift toward more ionic bonding in the glass network. This behavior is chemically reasonable, since samarium is a rare-earth element with pronounced metallic character; consequently, an increase in Sm_2_O_3_ concentration leads to a higher ionic character of the bonds within the glass network.

The optical band gap energy, E_g_, was obtained from the Kubelka–Munk function using the relation [F(R)hν]^2^ = k(hν − E_g_), where k is the proportionality constant, hν is the photon energy, and E_g_ is the optical band gap energy. E_g_ was estimated from the [F(R)hν]^2^ versus hν plots by extrapolating the linear region of each curve to the photon energy axis. As shown in Figure 5, the E_g_ values lie within a narrow range, varying from 4.29 to 4.36 eV. This small variation in $E_{g}$ across the studied composition range indicates that Sm_2_O_3_ addition has only a minor effect on the optical band gap of the studied glass system.

### 3.4. Luminescence Data

**Figure 6:**
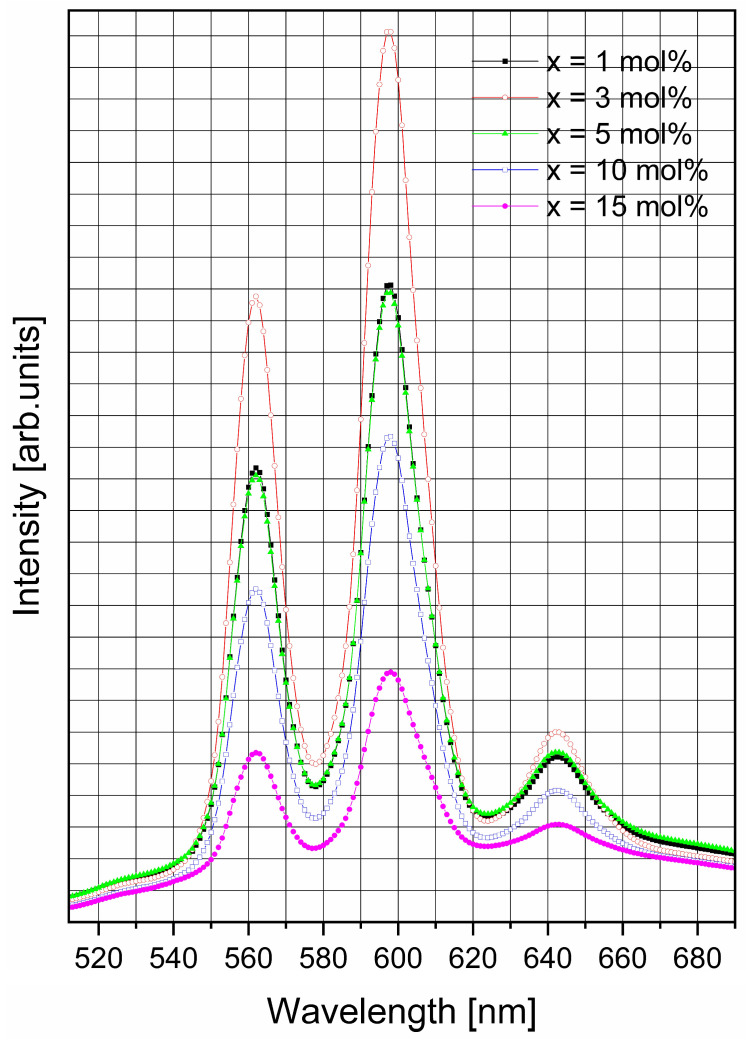
Luminescence spectra of the studied glasses.

Figure 6 presents the photoluminescent spectra of the samples of the xSm_2_O_3_·(100 − x)[2GeO_2_·2B_2_O_3_·Sb_2_O_3_·ZnO] glassy system. The spectra were recorded at room temperature under excitation at 402 nm, corresponding to the most intense band in the excitation spectra. Three characteristic emission bands associated with Sm^3+^ ions are observed, in agreement with the previous literature reports [6,39,40,41]. The emission band centered at 562 nm is assigned to the ^4^G_5/2_ → ^6^H_5/2_ transition, while the most intense band at 597 nm corresponds to the ^4^G_5/2_ → ^6^H_7/2_ transition. The band located at 643 nm is attributed to the ^4^G_5/2_ → ^6^H_9/2_ transition.

A closer examination of the spectra indicates that the positions of the emission bands remain essentially unchanged with increasing Sm_2_O_3_ concentration, and reveal that the local environment of the Sm^3+^ ions is only slightly affected by composition. In contrast, the emission intensity increases with samarium content up to 3 mol% Sm_2_O_3_, after which a decrease is observed due to concentration quenching. This luminescent behavior can be attributed to the reduced distance between neighboring Sm^3+^ luminescent centers at higher dopant concentrations, which enhances non-radiative energy transfer processes and consequently lowers the emission intensity.

The emission peak position does not change with the increase in Sm_2_O_3_ concentration in the batch. On the other hand, the emission intensity increases up to 3 mol% of Sm_2_O_3_ in the glass and after that decreases due to the quenching phenomenon.

The increased concentration (above 3 mol% of Sm_2_O_3_) leads to a higher number of luminescent centers and to a lower distance between them. Consequently, non-radiative processes appear, and the quenching of luminescent emissions occurs. Since the quenching phenomenon is observed starting in small concentrations of samarium, the quenching mechanism is Förster type via multipolar interactions; the distance between luminescent ions is larger (the ions are too far from each other).

On the other hand, in the case of Sm^3+^ ions, parts of transitions are allowed, so the dominant interactions are dipole–dipole type.

The luminescence results suggest the following emission mechanism. Under 402 nm excitation, Sm^3+^ ions are promoted from the ^6^H_5/2_ ground state to the ^6^P_5/2_ excited level, followed by non-radiative relaxation. Because the most intense emission band is situated around 597 nm (in the yellow–orange range of the visible spectrum) materials are suitable for yellow–orange-emitting solid-state laser applications.

The luminescence findings show that the intensity of the magnetic dipole emission (597 nm) is higher than electric dipole emission (643 nm), suggesting that the symmetry around the Sm^3+^ ions is higher (an octahedral one or a distorted octahedral), in this case samarium being surrounded by six oxygens [42,43].

**Figure 7:**
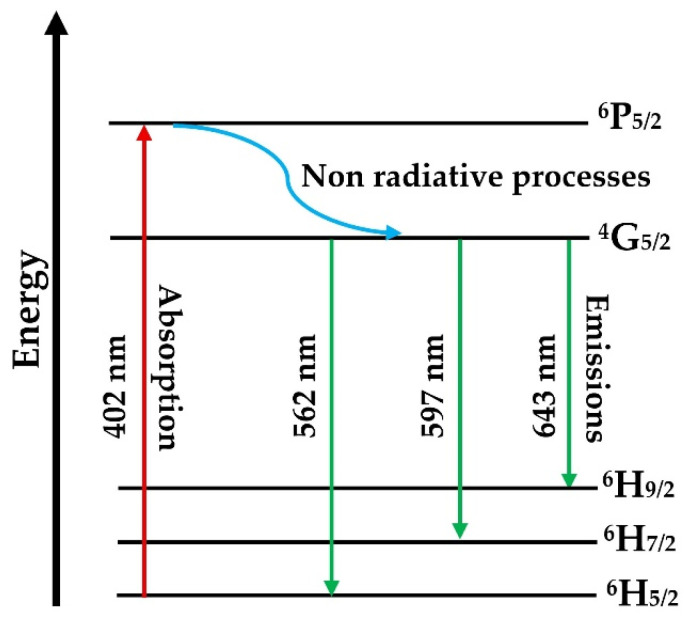
Energy-level diagram and cross-relaxation channels of the Sm^3+^ ion.

The energy-level diagram corresponding to the emission recorded under 402 nm excitation is presented below (Figure 7).

### 3.5. Excitation Spectra

**Figure 8:**
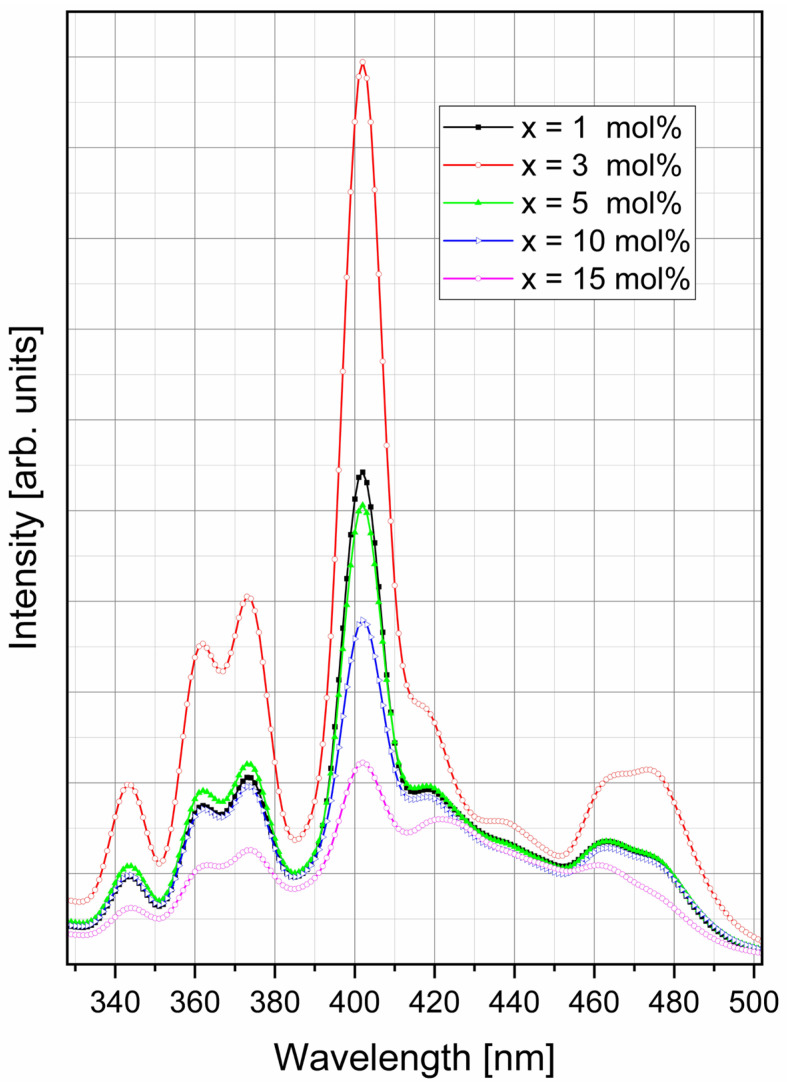
Excitation spectra of the xSm_2_O_3_·(100 − x)[2B_2_O_3_·2GeO_2_·Sb_2_O_3_·ZnO] glasses.

The excitation spectra of xSm_2_O_3_·(100 − x)[2GeO_2_·2B_2_O_3_·Sb_2_O_3_·ZnO] glasses, recorded at room temperature while monitoring the Sm^3+^ emission at 598 nm (Figure 8), show eight broad bands assigned to electron transitions from the ^6^H_5/2_ ground state to the ^4^H_9/2_ sau ^4^D_7/2_ (343 nm), ^4^D_3/2_ (362 nm), ^6^P_7/2_ (373 nm), ^6^P_3/2_ (402 nm), ^6^P_5/2_ (415 nm), ^4^G_9/2_ (435 nm), ^4^I_13/2_ (464 nm) and ^4^I_11/2_ (472 nm) excited states [4,5,7,8,9,10]. The broad appearance of these bands is characteristic of Sm^3+^-doped glasses, and is attributed to inhomogeneous broadening caused by the distribution of local environments around Sm^3+^ ions and by overlap between Stark components of the 4f–4f transitions. The band near 402 nm is the most intense for all compositions, demonstrating that the near-UV excitation is the most effective pathway for producing the orange emission (~598 nm).

Therefore, the 402 nm excitation was used for photoluminescence measurements of the Sm^3+^-doped zinc–antimony–boro–germanate glasses.

Figure 8 reveals a strong connection between concentration and excitation behavior. Thus, the sample containing 3 mol% Sm_2_O_3_ exhibits the highest excitation intensity over nearly the entire spectral range and, in particular, the most intense band at approximately 402 nm. In comparison, the samples with 1 and 5 mol% Sm_2_O_3_ display lower intensities, whereas those containing 10 and 15 mol% Sm_2_O_3_ exhibit a pronounced decrease. These results indicate that excitation efficiency increases with Sm_2_O_3_ concentration up to 3 mol% owing to the increasing number of optically active centers, but declines at higher concentrations as non-radiative energy transfer between adjacent Sm^3+^ ions becomes more significant. Such behavior is characteristic of concentration quenching and demonstrates that the zinc–antimony–boro–germanate glass matrix accommodates Sm^3+^ ions most effectively at intermediate concentrations, while higher dopant contents promote non-radiative loss processes.

It is also worth noting that the positions of the main excitation bands remain almost unchanged with increasing Sm_2_O_3_ concentration, whereas their intensities vary significantly. The absence of a clear systematic spectral shift suggests that the average local field strength around the Sm^3+^ ions is only weakly affected over the studied compositional range. Therefore, the concentration effect is mainly associated with the changes in the balance between radiative and non-radiative processes rather than with the changes in transition energies. This effect is commonly observed in multicomponent Sm^3+^-doped glasses, in which concentration-induced structural changes are strongly manifested in intensity and decay parameters and lesser in-band positions.

## 4. Conclusions

The XRD patterns of the studied samples reveal their amorphous state over the investigated concentration range.

The XPS results confirmed Sm ions incorporation into the zinc–antimony–boro–germanate oxide glasses and showed that samarium is predominantly stabilized in the trivalent state after synthesis, consistent with its function as the luminescent center.

UV–Vis measurements revealed that the undoped matrix does not exhibit distinct absorption bands, whereas the sample with the highest Sm_2_O_3_ concentration shows ten absorption bands assigned to intra-4f electronic transitions. The calculated bonding parameter further indicated that increasing Sm_2_O_3_ content enhances the ionic character of the bonds within the glass network.

Photoluminescence spectra exhibited three characteristic emissions bands that correspond to the Sm^3+^ luminescent center. The emission intensity increased up to 3 mol% Sm_2_O_3_, above which concentration quenching was observed. Since the most intense emission band is located in the yellow–orange region of the visible spectrum, the materials are appropriate for yellow–orange-emitting laser applications.

The excitation spectra demonstrated a pronounced concentration dependence of the optical response, with the highest excitation efficiency observed for the sample containing 3 mol% Sm_2_O_3,_ when monitoring the emission at 598 nm. The presence of a dominant excitation band near 402 nm, along with weaker bands in the blue region (approximately 462–472 nm), further confirms the suitability of these glasses for near-UV-pumped orange-emitting photonic devices.

## Data Availability

The original contributions presented in this study are included in the article. Further inquiries can be directed to the corresponding authors.
